# Optimizing support vector machine (SVM) by social spider optimization (SSO) for edge detection in colored images

**DOI:** 10.1038/s41598-024-59811-z

**Published:** 2024-04-21

**Authors:** Jianfei Wang

**Affiliations:** Suzhou Chien-Shiung Institute of Technology, Taicang, 215411 China

**Keywords:** Edge detection, Support vector machine, Social spider optimization, Image processing, Applied mathematics, Computational science, Computer science, Information technology

## Abstract

Edge detection in images is a vital application of image processing in fields such as object detection and identification of lesion regions in medical images. This problem is more complex in the domain of color images due to the combination of color layer information and the need to achieve a unified edge boundary across these layers, which increases the complexity of the problem. In this paper, a simple and effective method for edge detection in color images is proposed using a combination of support vector machine (SVM) and the social spider optimization (SSO) algorithm. In the proposed method, the input color image is first converted to a grayscale image, and an initial estimation of the image edges is performed based on it. To this end, the proposed method utilizes an SVM with a Radial Basis Function (RBF) kernel, in which the model's hyperparameters are tuned using the SSO algorithm. After the formation of initial image edges, the resulting edges are compared with pairwise combinations of color layers, and an attempt is made to improve the edge localization using the SSO algorithm. In this step, the optimization algorithm's task is to refine the image edges in a way that maximizes the compatibility with pairwise combinations of color layers. This process leads to the formation of prominent image edges and reduces the adverse effects of noise on the final result. The performance of the proposed method in edge detection of various color images has been evaluated and compared with similar previous strategies. According to the obtained results, the proposed method can successfully identify image edges more accurately, as the edges identified by the proposed method have an average accuracy of 93.11% for the BSDS500 database, which is an increase of at least 0.74% compared to other methods.

## Introduction

Edge detection in images is one of the fundamental and low-level topics in the field of image processing. The detected edges and boundaries of objects can be used for various applications such as object recognition^1^, image editing^2^, image segmentation^3^, and so on. Therefore, improving edge detection techniques can enhance a wide range of applications in the field of machine vision. The goal of edge detection methods is to distinguish prominent changes in pixel brightness that manifest as discontinuities in intensity, color, or texture^4^. Initial edge detection methods used information related to gradients or first and second-order derivatives to identify the boundaries of image edges^5^. Although these strategies were suitable for initial edge approximation and not highly accurate, they still enjoyed high popularity. With the development of machine learning techniques and subsequently deep learning techniques, newer strategies have been proposed for edge detection in images. These methods can identify edge boundaries in images based on previously observed patterns^6^.

Machine learning-based techniques generally exhibit higher accuracy compared to initial methods. For this reason, in recent years, we have witnessed numerous methods for edge detection in images using these techniques. However, research in this field faces two challenges. Firstly, most of the proposed research focuses on edge detection in grayscale images, neglecting the color features of the image^7^. Secondly, the employed learning models in many of these studies cannot guarantee the highest achievable accuracy by the model. Based on these considerations, this research presents a color image edge detection model based on the combination of machine learning and optimization techniques. The proposed method in this paper is a two-stage edge detection strategy, where an optimized SVM model using the SSO algorithm is used for the initial approximation of image edges. Additionally, a color-based edge enhancement technique is employed in this method, which can improve the accuracy of edge detection in color images. The contributions of this paper are as follows:

- In this paper, an optimized machine learning model based on SVM with a RBF kernel is presented for edge detection in images. The SSO optimization algorithm is utilized to fine-tune the hyperparameters of SVM, leading to edge detection with lower error compared to conventional models.
- A two-stage model for edge detection in color images is proposed in this research. In the first stage, image edges are estimated based on the grayscale image, and then, in the second stage, edge improvement is performed through matching the estimated edges with pairwise combinations of color image layers.
- The proposed model in this research is implemented using GPU array processing technology, and the effectiveness of this process on enhancing the speed of image edge detection is evaluated.

The continued structure of the current article is as follows: "Literature review" includes a review of previous research. In "Proposed method", the details of the proposed two-stage strategy for edge detection in color images are presented. Then, in "Results and discussion", the evaluation and research results are discussed. "Conclusion" provides a summary of the findings and proposes suggestions for further research in this field.

## Literature review

Edge detection is a fundamental step in image processing with applications in various domains, including satellite image processing^8^, object detection^9^, asymmetric image processing^10^, and medical image analysis^11^. While a significant amount of research has focused on edge detection in grayscale images, color images present a more complex challenge due to the combined information from multiple color channels^12^. This section reviews recent advancements in edge detection for color images, categorizing them into different approaches and discussing their advantages and limitations.

### Frequency-domain methods

Some approaches utilize frequency-domain analysis for edge detection. Bhatti et al.^8^ propose a method for satellite image edge detection using Clifford algebra and Quaternion Fourier Transform (QFT). This method leverages frequency domain information from each color channel to identify edges. However, the computational complexity of QFT can be a limitation.

### Gradient-based methods

Traditional edge detection techniques often rely on gradients to identify image discontinuities. The Canny edge detector^9^ is a widely used example, employing a multi-stage approach to achieve good edge localization and noise suppression. However, the Canny operator requires careful parameter tuning for optimal performance, and its effectiveness can vary depending on the color space used^12^.

### Fuzzy-logic based methods

Fuzzy logic offers an alternative approach to edge detection. Orujov et al.^11^ present a fuzzy method for detecting artery edges in retinal images that solely utilizes information from the green channel. This method demonstrates promising results in specific applications but may not be suitable for general-purpose color image edge detection.

### Deep learning techniques

Deep learning has emerged as a powerful tool for image processing tasks, including edge detection. Soria et al.^13^ propose a deep convolutional neural network (CNN) architecture specifically designed for edge detection in color images. This approach achieves high accuracy but requires significant training data and computational resources. Another deep learning-based method by Wang et al.^14^ focuses on apple edge detection for monitoring fruit growth. This method highlights the ability of deep learning to handle specific edge detection tasks in color images. However, deep learning models often require careful design and optimization to achieve optimal performance. In^15^, a deep learning model for edge detection in color images is presented, inspired by the combination of the Holistically-Nested Edge Detection (HED) method and Xception networks. The proposed deep neural network model in this research is similar to the model presented in^13^. Similarly, this model also consists of 6 convolutional blocks, and the output of each block is enhanced by an upsampling block. In this research, a new database is also used for training the convolutional model in a similar manner. In general, deep learning techniques due to their high performance in image processing have been used in various applications such as image classification^16^, image reconstruction^17^ or synthesizing photo-realistic images^18^; and the application areas of these models are increasing^19^.

### Other techniques

Several other techniques have been explored for edge detection. Versaci et al.^20^ propose a method based on fuzzy entropy and fuzzy divergence for capturing image edge information. Peng et al.^21^ present a solution for metro path detection that combines the Canny operator with Hough transform for edge and line detection. Liu et al.^22^ utilize statistical features of edge ratios for edge detection, while research by Gandhi et al.^10^ explores different preprocessing strategies for improving edge detection results. This research presents a preprocessing strategy for asymmetric image processing, which can be effective in edge detection applications. Three preprocessing solutions are investigated in this method. In the first approach, after converting the image to grayscale, image noise is reduced using a Gaussian filter, and then a threshold is applied to convert the image into a binary matrix and approximate regions based on it. In the second approach, image noise is reduced using a Gaussian filter, and after converting the image to a grayscale matrix, a dual threshold is used to approximate regions in the image. In the third strategy, after converting the image to grayscale, a dual threshold is applied to approximate the edges, and finally, a Gaussian filter is used to remove noise. According to the results, the first and second approaches do not yield satisfactory results, while the third method can be suitable for approximating edges in images.

### Computational efficiency considerations

As image processing tasks become increasingly complex, computational efficiency is a crucial factor. Horvath et al.^23^ demonstrate significant speed improvements in the Canny operator by utilizing CUDA for parallel processing on GPUs. Similarly, Livingston et al.^24^ explore parallel processing techniques to enhance the speed of edge detection algorithms.

Edge detection in color images remains an active area of research. This review has presented various approaches, including traditional gradient-based methods, frequency-domain techniques, fuzzy logic-based approaches, deep learning models, and other techniques. Each approach offers advantages and limitations, and the most suitable method depends on the specific application and desired outcome. Future research directions may involve further exploration of deep learning architectures tailored for color image edge detection, as well as the development of more efficient and robust algorithms for real-time applications. Table 1, summarizes the literature review.

**Table 1:** Summary of the literature review.

Reference	Year	Purpose	Method	Limitation
Bhatti et al.^8^	2021	Edge detection in satellite color images	Clifford algebra and QFT	Computationally expensive
Mittal et al.^9^	2019	Improve edge connectivity and thickness	Multi-threshold Canny operator	Requires careful parameter tuning
Gandhi et al.^10^	2020	Preprocessing for edge detection	Gaussian filtering, thresholding	Limited effectiveness for complex images
Orujov et al.^11^	2020	Detect artery edges in retinal images	Fuzzy logic with green channel information	Not suitable for general-purpose color image edge detection
Ismael et al.^12^	2020	Compare performance of Prewitt operator in different color spaces	Prewitt edge detection	Performance varies depending on color space
Soria et al.^13^	2021	Dense extreme inception network for edge detection	Deep CNN	Requires significant training data and computational resources
Wang et al.^14^	2020	Deep learning for apple edge detection	Deep CNN	Requires careful design and optimization for specific tasks
Poma et al.^15^	2020	Dense extreme inception network for edge detection	Deep CNN	Similar limitations as^13^
Versaci et al.^20^	2021	Image edge detection based on fuzzy entropy and fuzzy divergence	Fuzzy entropy and fuzzy divergence	Limited research on general applicability
Peng et al.^21^	2023	Metro path detection	Canny operator and Hough transform	Not specifically designed for general-purpose color image edge detection
Liu et al.^22^	2020	Edge detection based on edge ratio statistics	Statistical features of edge ratios	May not be suitable for all types of edges
Horvath et al.^23^	2023	Improve speed of Canny operator	CUDA for parallel processing on GPUs	Limited to Canny operator
Livingston et al.^24^	2019	Enhance speed of edge detection algorithms	Parallel processing techniques	Limited details on specific improvements for color images

## Proposed method

The proposed method for edge detection in color images is performed at two levels. At the first level, image edges are approximated through a SVM optimized by the SSO algorithm using the grayscale image. Then, at the second level, the SSO algorithm is used to improve the approximated edges based on the comparison with pairwise combinations of color layers in the original image. It should be noted that the proposed method assumes that the input image is presented in RGB color system. Based on this, the steps of the proposed method for edge detection in color images are as follows:

1. Edge approximation based on SVM and SSO.
2. Edge improvement based on SSO and the difference with color layer combinations.

**Figure 1:**
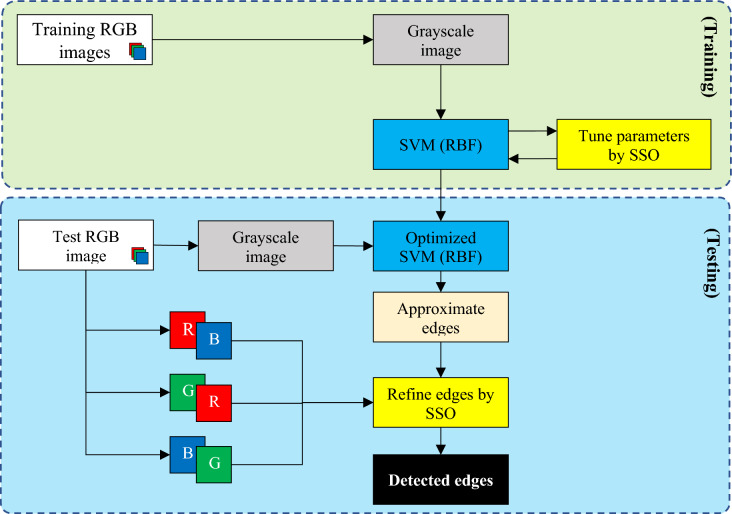
Stages of edge detection in color images in the proposed method.

The architecture of the proposed method is illustrated in Fig. 1 as a diagram. In this figure, the steps related to each phase of training (SVM model optimization) and testing (edge detection in new images) are separated from each other.

The proposed method begins with optimizing the SVM model based on the training samples of the database using the SSO algorithm. For this purpose, the RGB training images are first converted to the grayscale color system, and then these samples, along with their ground-truth edges, are used as the input training data for the SVM model. The proposed SVM model utilizes a RBF kernel function, and its parameters are optimized using the SSO algorithm. The goal of this process is to achieve an SVM model with the least error in approximating the edges of the grayscale image. The result of this process is an SVM model with the best discovered configuration by the SSO algorithm, which is used for the initial approximation of edges in test images. The approximated edges identified through this model are compared with pairwise combinations of color layers, and an effort is made to minimize the difference between the edge pixels and the identified boundaries in different layer combinations using the SSO algorithm. The outcome of this process is the detected edges in the image.

### Edge approximation based on SVM and SSO

**Figure 2:**
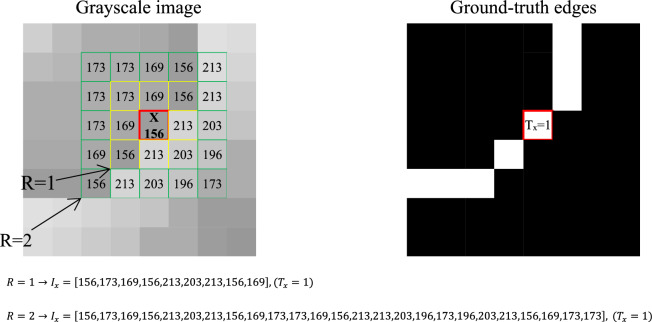
The process of a training record formation for a sample pixel in the proposed method.

The proposed method starts with the initial approximation of image edges using the combination of SVM and SSO. For this purpose, a training set of RGB images is used, where for each RGB image, there is a binary matrix indicating the background edges of the image. This training set is used as the training samples for the SVM model. At the beginning of this stage, each RGB image is first converted to the grayscale color system. Then, the training records are formed based on the pixel values present in the grayscale image. To do so, the intensity information of the current pixel and the intensity of pixels within the neighboring radius R are extracted. By transforming these values into a vector form, a training record for the SVM model is constructed. Additionally, the corresponding value for each pixel in the ground-truth edge image is considered as the target label for that training sample. Figure 2 illustrates the process of forming a training record for a sample pixel. In this figure, the assumed pixel is represented as x. Assuming R = 1, the length of the feature vector will be 9, and if R = 2, then the features of each pixel will be described in a vector form with a length of 25. It should be noted that, considering the learning properties of the SVM model, the order of features does not affect the final result. Also, for pixels on the image boundary that fall outside the image extent, the values of their missing neighboring pixels are set to zero during training data generation.

After forming the training records based on the training RGB images and their ground-truth edges, the SVM model is trained and optimized. The proposed method utilizes an SVM model with a RBF kernel as an initial estimator for the image edges. SVM, a well-known binary classifier, has been widely used in various problem-solving scenarios. This classifier attempts to create a boundary that separates the samples of the two target classes. By creating this boundary, the samples of each class will reside in a region known as the hyperplane. The margin, which represents the minimum distance between the samples and the boundary between the hyperplanes, is considered. The objective of the SVM training algorithm is to maximize the margin between the hyperplanes^25^.

Since the relationship between the input variables (pixel intensity and its neighbors) and the target classes (pixel membership in an edge) in the discussed problem is nonlinear, a non-linear kernel function called the RBF has been used to solve this problem with SVM. The radial basis function kernel is formulated as follows in the SVM model^26^:

1 $$k\left({x}_{i}.{x}_{j}\right)={\text{exp}}(-\gamma {\left|\left|{x}_{i}-{x}_{j}\right|\right|}^{2})$$

In the above relationship, ${x}_{i}.{x}_{j}$ represents the dot product of the feature vectors for samples ${x}_{i}$ and ${x}_{j}$. This is a common kernel trick used in SVMs with RBF kernels. Moreover, γ represents the coefficient of the kernel function. On the other hand, the number of samples belonging to the two classes, positive (edge members) and negative (background members), is unbalanced. This imbalance can lead to a decrease in training quality and an increase in errors. To address this issue in the SVM model, a correction parameter is adjusted separately for each class. In this case, the optimization problem of the SVM model can be described as follows^26^:

2 $$\begin{array}{c}minimiz{e}_{w,b}\frac{1}{2}\left|\left|w\right|\right|+{C}_{+}\sum_{{d}_{i}=1}{\xi }_{i}+ {C}_{-}\sum_{{d}_{i}=-1}{\xi }_{i} \\ subject\;to\;{D}_{i}\left(w.{x}_{i}+b\right)\ge \varepsilon -{\xi }_{i}, {\xi }_{i}\ge 0\end{array}$$

In the above relationship, w represents the normal vector of the hyperplane, and b determines the margin coefficient. Additionally, ${x}_{i}$ represents the i-th training sample, and ${d}_{i}$ describes the corresponding label for that sample. Moreover, ${\xi }_{i}$ represents the slack variable. For a sample i which has been correctly classified ${\xi }_{i}=0$ and otherwise, ${\xi }_{i}$ is the distance between i and its hyperplane. Finally, ${C}_{+}$ and ${C}_{-}$ are the correction parameters for the positive and negative classes, respectively.

For problems with balanced number of samples in target classes, the correction parameters for the positive and negative classes can be considered the same. But, the edge detection task is a highly imbalanced problem that the number of samples of the positive category (edge pixels) is insignificant compared to the number of samples of the negative category (background pixels). For this reason, the correct performance of the SVM model depends on the precise setting of the correction parameters for the positive and negative classes. Accordingly, an SVM model can be optimized using the correction parameters ${C}_{+}$ and ${C}_{-}$, as well as the radial basis coefficient γ. This process can be formulated as an optimization problem that utilizes the SSO algorithm in the proposed method.

Next, the defined structure for each solution vector and the evaluation procedure for objective function will be presented. Then, the optimization steps of the SVM model based on the SSO will be described.

As mentioned, in the process of configuring the SVM model using SSO, the goal is to determine the optimal values for the parameters ${C}_{+}$,${C}_{-}$, and γ. Each of these parameters is considered as an optimization variable that can take real values. Therefore, the length of each solution vector will be 3. The search bounds of each optimization variable has been determined experimentally. In each solution vector, the search bounds for ${C}_{+}$ and ${C}_{-}$ are set as $\left[{10}^{-2},3.6\times {10}^{4}\right]$. This bound, covers all possible valid combinations of hyperparameters ${C}_{+}$ and ${C}_{-}$ in edge detection problem. On the other hand, the search bounds for the γ parameter are set as $\left[{10}^{-10},{10}^{2}\right].$

Defining the objective function can be considered as the key component in solving optimization problems. Because based on the objective function, the quality of a solution can be determined. In the proposed method, the objective function is defined based on a validation error criterion. Thus, to evaluate the objective value of each solution vector (the optimality of values specified for SVM hyperparameters), first, the considered values for hyperparameters ${C}_{+}$,${C}_{-}$, and γ are extracted from the solution vector and these values are applied to the SVM model. This, will result in a configured SVM model based on the parameters specified in that solution vector. Then, the configured SVM model is trained on the training data. Finally, by applying the validation samples to the trained model, the training error is considered as the objective function.

3 $$Objective=\frac{E}{N}$$

In the above equation, E represents the number of misclassified records in the validation set. In other words, it signifies the number of validation samples for which the SVM model, configured with the parameters from a specific solution vector, produced an output class different from the actual class label. Additionally, the parameter N represents the total number of records in the validation set.

Minimizing this objective function value is the goal of the SSO algorithm. A lower value indicates better performance of the SVM model on unseen data (validation set) and translates to a more accurate edge detection outcome. The basic idea of SSO algorithm is using the cooperative behavior of social spiders in colony. This algorithm works based on search agents that are modeled as spiders. Unlike most swarm intelligence methods where search agents have the same behavior; In SSO, two types of search agents (male and female spiders) with different behavior are defined, which can lead to a more efficient approach of searching the problem space. The SSO algorithm starts by initializing the population. Then an iterative mechanism is used for finding the optimal solution. At the beginning of each search cycle, the objective value of each spider is calculated. Then, based on the calculated weight, a weight value is assigned to each spider which can model the attractiveness of the spider for others^27^:

4 $${w}_{i}=\frac{Objectiv{e}_{i}-worst}{best-worst}$$

In the above equation, the term $fitnes{s}_{i}$ represents the calculated objective value for solution i, while "worst" and "best" indicate the worst and best objective values in the current population, respectively. The weight of each spider is used to calculate the vibration level of each spider as follows^27^:

5 $${V}_{\left(i,j\right)}={w}_{j}{e}^{{d}_{i,j}^{2}}$$

In the above equation, ${w}_{j}$ represents the weight assigned to spider $j$ according to Eq. (4), and $d$ represents the distance between two spiders. It should be noted that each spider, like $i$, accepts only three types of vibrations. The first type is represented as $n$ and indicates vibrations generated by a closer spider with a higher weight. The second type represents vibrations generated by a female spider and is only accepted by male spiders. Finally, the third type represents vibrations generated by the best spider in the population. In the next step of SSO cycles, the position of each spider is updated. SSO uses two different strategies for simulating the movement pattern of male and female spiders. The position of female spiders is updated based on their current position and received vibrations as follows^27^:

6 $${f}_{i}\left(k+1\right)=\left\{\begin{array}{c}{f}_{i}\left(k\right)+\alpha .{V}_{i,n}.{(s}_{n}-{f}_{i}(k))+\beta . {V}_{i,b}.({s}_{b}-{f}_{i}(k))+\delta .(rand-0.5)\;with\;{P}_{f} \\ {f}_{i}\left(k\right)-\alpha .{V}_{i,n}.{(s}_{n}-{f}_{i}(k))-\beta . {V}_{i,b}.({s}_{b}-{f}_{i}(k))+\delta .(rand-0.5)\;with\;{1-P}_{f}\end{array}\right.$$

In the above equation, the parameters $\alpha$, $\beta$, and $\delta$ are random numbers in the range [0, 1]. Also, ${s}_{n}$ and ${s}_{b}$ represent the position of the neighboring and the best spider, respectively. Finally, ${f}_{i}(k)$ represents the current position of spider i, and ${f}_{i}\left(k+1\right)$ describes its new position. On the other hand, the position of male spiders is updated by considering the vibration from female spiders, also^27^:

7 $${m}_{i}\left(k+1\right)=\left\{\begin{array}{c}{m}_{i}\left(k\right)+\alpha .{V}_{i,f}.{(s}_{f}-{m}_{i}\left(k\right))+\delta .(rand-0.5) \quad if \, {m}_{i}\left(k\right) \, is \, dominant \\ {f}_{i}\left(k\right)-\alpha . \left(\frac{\sum_{j\in ND}{m}_{j}\left(k\right).{w}_{j}}{\sum_{j\in ND}{w}_{j}}- {m}_{i}\left(k\right)\right) \quad if\, {m}_{i}\left(k\right)\, \;is\, \;nondominant\end{array}\right.$$

In the above equation, ${s}_{f}$ represents the position of the nearest female spider. At the final step of each cycle in SSO, the mating and survival operators are applied to the population. If a male spider is dominant, it can perform a mating operation with female spider within range r. This will result in a new solution such as ${C}_{new}$. In order to simulate the survival operation for ${C}_{new}$, its objective value is compared with worst. If ${C}_{new}<worst$, then the new solution is replaced with the worst solution in the population; otherwise, ${C}_{new}$ is discarded. The described cycle is repeated for a predetermined number of times. In the following, the pseudo code of proposed procedure for SVM optimization by SSO algorithm is presented.

**Algorithm 1:**
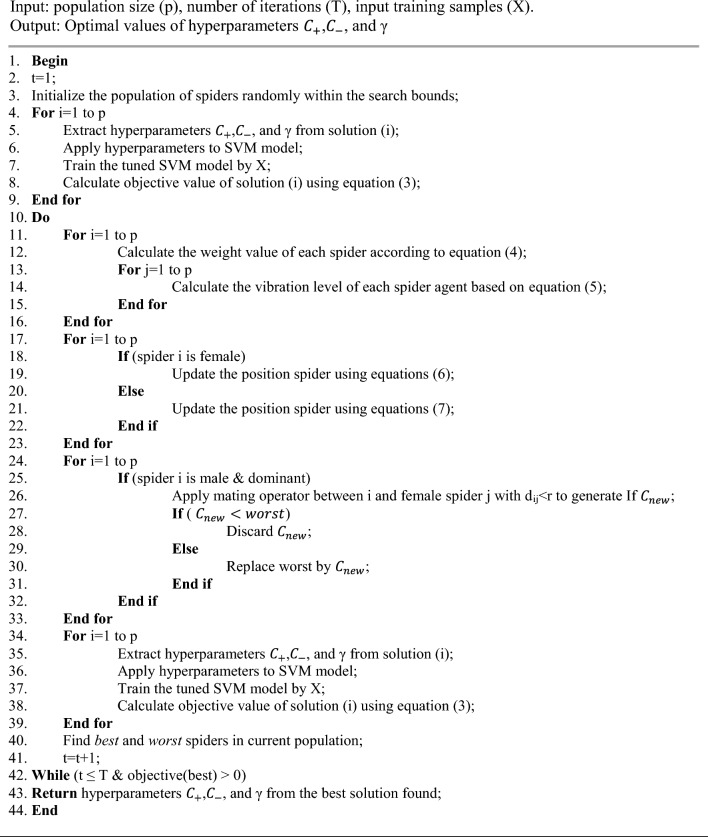
SVM optimization by SSO.

After executing the above steps, the determined configuration in the solution vector with the minimum objective value is applied to the SVM model, and this model is used for the initial estimation of edges in the test samples. To do this, the test image is first converted to a grayscale system, and then the feature vectors of each pixel in the image are formed based on the process described at the beginning of this section. By feeding these vectors to the optimized SVM model, the membership of each pixel in the image to the edge region is determined. The binary matrix resulting from the aggregation of the SVM model outputs is considered as the initial approximation of the edge map of the grayscale image.

### Edge enhancement based on SSO and difference with color channels combinations

**Figure 3:**
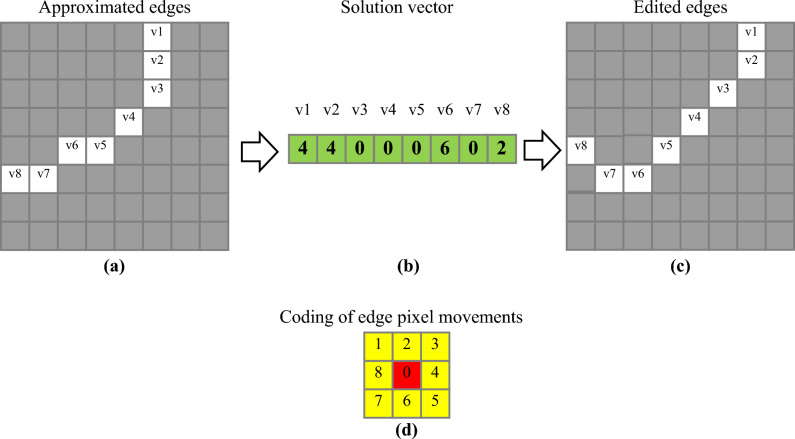
An example of edge improvement process using the SSO algorithm.

After creating the edge approximation matrix, the SSO algorithm is used to improve the edge regions in the image. The search steps for finding the optimal solution by the SSO algorithm in this step are similar to the previous step, with the difference that a different structure is used for encoding the solution vector and evaluating its objective value. This structure is explained through an example. Consider an edge approximation matrix (result of the previous step) as shown in Fig. 3a. The SSO algorithm in this step attempts to match each pixel on the edge with the intensity values of the three matrices L_1_, L_2_, and L_3_ by displacing them. In this case, each edge pixel is considered as an optimization variable that can be moved by at most one pixel in the solution vector. In Fig. 3a, each edge pixel is represented by optimization variables v_1_ to v_8_. Thus, for this sample image, the length of the solution vector will be 8. An example solution vector for this example is shown in Fig. 3b. Each element of this vector corresponds to one of the optimization variables (edge pixels v_1_ to v_8_), and the value in each position of the solution vector determines the displacement of that edge pixel. The encoding scheme for the solution vector for each pixel is shown in Fig. 3d. According to this figure, the number 0 indicates no displacement of the edge pixel, and numbers 1 to 8 indicate displacement in one of the surrounding directions with a radius of 1. By applying the solution vector shown in Fig. 3b to the edge matrix in Fig. 3a, the resulting image will be as shown in Fig. 3c, where pixels v_1_, v_2_, v_6_, and v_8_ are displaced according to the assumed solution vector pattern.

With these explanations, each solution vector of the SSO algorithm in the second step of the proposed method will have a length equal to the number of pixels located on the edge in the initial approximation image. This length indicates the number of optimization variables, and each optimization variable is described by an integer in the range [0, 8].

After editing the edges based on the solution vector, the fitness evaluation is performed by comparing the values of edge pixels in pairwise combinations of color layers in the original image. For this purpose, each pairwise combination of color layers, in the form of <RG, GB, BR> , is transformed into a matrix using the following equation:

8 $${L}_{c}=\frac{1}{2}\times \left({I}_{1}+{I}_{2}\right), {I}_{1},{I}_{2}\in \left\{R,G,B\right\}, {I}_{1}\ne {I}_{2}$$

In the above equation ${I}_{1},{I}_{2}$ represent two different layers of the RGB image, and *Lc* represents the uniform combination of these two layers. By applying the above equation to the combinations of <RG, GB, BR> layers, three matrices, denoted as L_1_, L_2_, and L_3_, will be obtained, corresponding to the combination of RG, GB, and BR layers, respectively. In this case, the fitness evaluation of the solution vector S is performed using the following equation:

9 $$Fitness\left(S\right)=\frac{1}{1+\frac{1}{3|S|} \sum_{i=1}^{3}\sum_{j=1}^{|S|}std({L}_{i}^{j}\cup N\left({L}_{i}^{j}\right))}$$

In the above equation, ${L}_{i}^{j}$ represents the edge pixel value in the layer ${L}_{i},(i=\mathrm{1,2},3)$ and $N\left({L}_{i}^{j}\right)$ denotes the sum of values of its neighboring pixels (within a radius of 1). Additionally, the standard deviation function is shown as $std\left(.\right)$. Finally, |S| represents the number of optimization variables or the number of edge pixels. According to the equation, to evaluate the fitness of each solution vector, the edge pixels are first moved according to the displacement vector, and then the mean standard deviation at the positions corresponding to the edge pixels in the combined layer combinations L_1_, L_2_, and L_3_ is calculated. By performing this process, we can ensure that the edge pixels are located on the borders of the regions in the color layers of the initial image, and the maximum distinction between the color regions can be achieved based on the edges. Since the goal of the optimization algorithm is to maximize the mean standard deviation, Eq. (9) is formulated in an inverse form, and the addition of 1 is included to prevent division by zero (for completely uniform regions in the images). With these explanations, the SSO algorithm in the second step of the proposed method attempts to edit the positions of the edge pixels in the initial approximation in a way that minimizes Eq. (9). The pattern obtained from the optimal solution vector in this step will be applied to the edge approximation matrix to obtain the output of the proposed method based on it.

## Results and discussion

In order to implement the proposed method, MATLAB 2019a software has been used. All tests were performed on a personal computer, running 64-bits version of windows 10 on an Intel core i7 processor with a processing power of 3.8 GHz and 32 GB of RAM. Also, the proposed model in this research has been implemented using array processing technology in graphical memory, in which an NVIDIA RTX 2080 Ti graphics adapter is used. During implementation, the processes of data record extraction and edge improvement in the second step of the proposed method have been implemented using graphic processors. In this case, the image is divided into 64 non-overlapping parts and the improvement of the edges of each part is performed by a separate process. Also, during the implementation, the Berkley Segmentation Dataset 500 (BSDS500)^28^ has been used.

### Database and implementation scenario

For the implementation of the proposed method and evaluating its performance in edge detection, the samples from the BSDS500 database have been used. This database consists of three sets of images: a training set with 200 samples, a validation set with 100 samples, and a testing set with 200 samples, collected for the purposes of segmentation and edge detection. All images are stored in the RGB color system and have different dimensions. Each sample in the BSDS500 database contains five ground truth edge annotations. During the experiments, the proposed model is trained based on the training set, specifically using Set 1. The SVM model employed in the proposed method is trained using the features of the training samples from the database, and then, for the optimization of the SVM model, the features of the validation samples are utilized with the SSO algorithm. Finally, in the testing phase, the performance of the proposed method is evaluated using the testing samples.

The BSDS500 training image set consists of more than 30.8 million pixels, with 506,113 pixels belonging to the image edges. Due to the highly unbalanced distribution of samples in the target classes, for training the SVM model, records corresponding to all edge pixels and 5% of the records corresponding to non-edge pixels have been used. The result of this process is 2,024,800 training records, which are reduced to 1,973,844 records after removing duplicate records. In this training set, there are 463,915 records for edge pixels (positive class) and the remaining records are for non-edge pixels (negative class). Repeating this process for validation samples resulted in the formation of 720,985 validation records, with 201,163 records belonging to the positive class and the rest belonging to the negative class. Finally, the test image set consists of more than 30.88 million pixels, all of which are evaluated by the proposed method for edge regions. After determining the image edges using the proposed method, each pixel can fall into one of the following four categories:

1. TP (true positive): represents the set of edge pixels that have been correctly detected by the edge detection algorithm.
2. TN (true negative): represents the set of non-edge pixels that the algorithm has correctly identified as not being part of an edge.
3. FP (false positive): indicates the set of non-edge pixels that have been mistakenly identified by the algorithm as edges.
4. FN (false negative): describes the set of edge pixels that the algorithm was unable to detect.

By measuring the four aforementioned sets, the performance of the algorithm in detecting image edges can be described based on precision, recall, and F-Measure metrics. The precision metric indicates the algorithm's effectiveness in correctly identifying edge pixels and shows the proportion of correct positive outputs of the algorithm. On the other hand, the recall metric indicates the algorithm's ability to correctly identify the proportion of ground-truth edge pixels. Finally, the F-Measure metric describes the overall performance of the algorithm in edge extraction by calculating the harmonic average of precision and recall. These metrics are formulated as follows:

10 $$Precision=\frac{TP}{TP+FP}$$

11 $$Recall=\frac{TP}{TP+FN}$$

12 $$F-Measure=2\times \frac{Precision\times Recall}{Precision+Recall}$$

Furthermore, the similarity between the edge-detected images by the proposed method and the ground truth images has been measured using structural similarity (SSIM), peak signal-to-noise ratio (PSNR), and mean squared error (MSE) metrics. The SSIM metric indicates the structural similarity between the detected edges by the proposed algorithm and the ground truth edges. In the ideal case, the structural similarity between the detected edges and the ground truth edges is maximum. The SSIM value varies between 0 and 1. Thus, in the best case, the SSIM value is 1, and in the worst case, it is 0. The SSIM metric can be calculated using the following formula^29^:

13 $$SSIM\left(Q,{Q}_{0}\right)=\frac{(2{\mu }_{Q}{\mu }_{{Q}_{0}}+{c}_{1})(2{\sigma }_{Q{Q}_{0}}+{c}_{2})}{({\mu }_{Q}^{2}+{\mu }_{{Q}_{0}^{2}}+{c}_{1})({\sigma }_{Q}^{2}+{\sigma }_{{Q}_{0}}^{2}+{c}_{2})}$$

where in the above equation, ${\sigma }_{Q}$ represents the root mean square variance of the original image Q, and ${\sigma }_{{Q}_{0}}$ represents the root mean square variance of the result image. ${\mu }_{Q}$ and ${\mu }_{{Q}_{0}}$ respectively indicate the mean intensity of the original and result images. Also, ${\sigma }_{Q{Q}_{0}}$ represents the root squared correlation among Q and Q_0_. In this equation, ${c}_{1}={\left({k}_{1}L\right)}^{2}$ and ${c}_{2}={\left({k}_{2}L\right)}^{2}$ are constant terms of the similarity index; where the values of k_1_, k_2_, and L are chosen to be 0.01, 0.03, and 255 respectively. The PSNR metric indicates the ratio between the maximum possible power of the signal (groung-truth edges) and the power of the noise (edge detection error). This metric can be calculated using the following formula^29^:

14 $$PSNR=10\times {{\text{log}}}_{10}(\frac{{255}^{2}}{MSE})$$

In the above equation, MSE represents the mean squared error. The goal of edge detection algorithms is to achieve a higher PSNR value. Finally, the MSE metric can be calculated as follows:

15 $$MSE=\frac{1}{N}\sum_{i=1}^{N}{\left({e}_{i}-{g}_{i}\right)}^{2}$$

where, N represents the number of pixels in the images, and ${e}_{i}$ and ${g}_{i}$ denote the pixel values at position i in the edge detection and ground-truth images, respectively.

## Results

**Figure 4:**
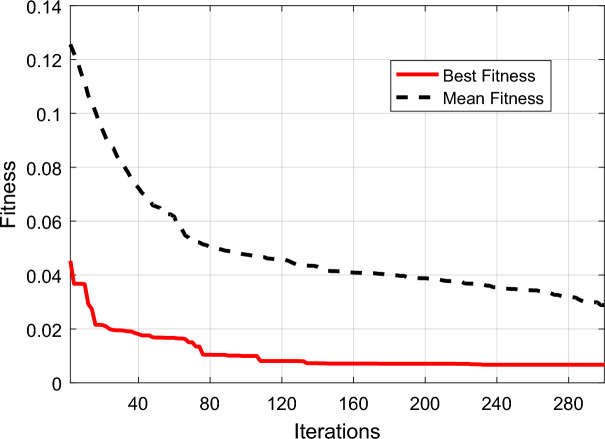
Convergence plot of the average fitness and best fitness discovered for SVM parameter optimization by SSO.

In order to implement the proposed method, MATLAB 2019a software was used. According to the procedure described in the previous section, training and validation samples were used to build and optimize the SVM model, and test samples were used to evaluate its performance. In the SVM model optimization phase, the population size and number of iterations of the SSO algorithm were set to 150 and 300, respectively. Additionally, in the edge improvement phase, these two parameters in the SSO algorithm were set to 200 and 400, respectively. Figure 4 shows the convergence plot of the SSO algorithm for optimizing the SVM model parameters. The plot demonstrates that by utilizing the SSO algorithm, the validation error can be reduced to less than 0.01. This optimal configuration was discovered in the 239th iteration of the SSO algorithm, and based on it, setting the parameters ${C}_{+}$, ${C}_{-}$ and $\upgamma$ to the values of 1.37, 15.89, and 0.0021, respectively, can result in the minimum validation error.

During the experiments, the performance of the proposed method was studied in two scenarios, R = 1 and R = 2. In the R = 1 scenario, the feature vector length for describing the attributes of each pixel is set to 9, while in the R = 2 scenario, the attributes of each pixel are described using 25 features (see Fig. 2). Additionally, to evaluate the effectiveness of each employed technique in the proposed method, the following cases were also studied:

- Proposed (without SSO): in this case, the SVM model optimization step using the SSO algorithm is ignored, and an SVM model with the RBF kernel function is used for the initial approximation of the image edges.
- Proposed (without L2): in this case, the edges approximated by the SVM model are considered as the final edges, and the edge refinement process using the SSO algorithm in the second step of the proposed method is ignored.

**Figure 5:**
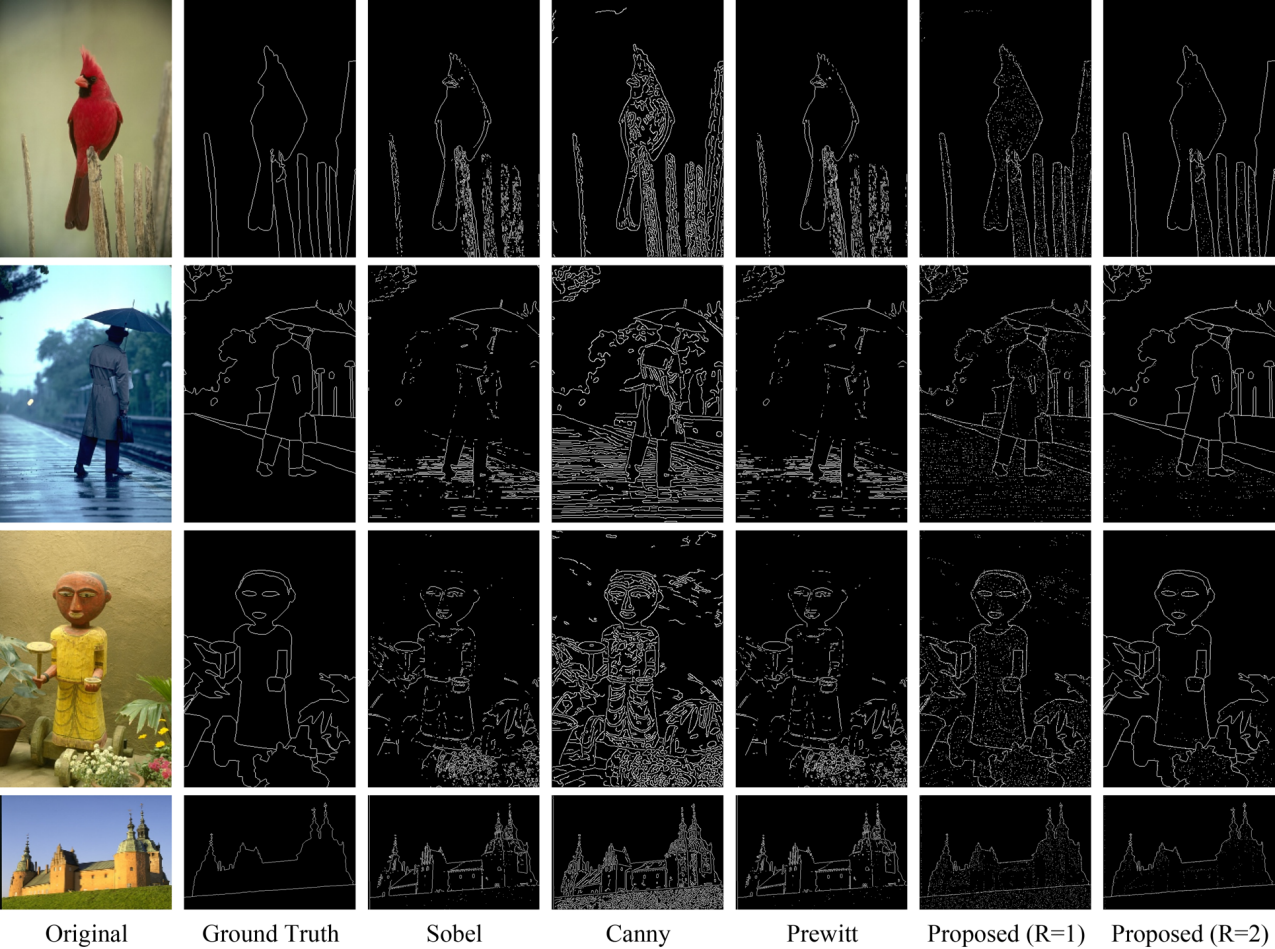
Several examples of edge-detected images using the proposed method and other methods from the BSDS500 database.

It should be noted that in both of the above cases, the neighborhood radius parameter is set to R = 2. Furthermore, the performance of the proposed method is compared with the methods of Soria et al.^13^ and Poma et al.^15^. In Fig. 5, several examples of edge-detected images using the proposed method from the BSDS500 database are displayed. In this figure, in addition to the output of the proposed method, the results of edge detection by the Canny, Prewitt, and Sobel algorithms are also shown.

As the results presented in Fig. 5 demonstrate, the edges identified by the proposed method are closer to the ground truth images. Comparing the results of the proposed method with the Canny, Prewitt, and Sobel edge detectors shows that the performance of the proposed method is clearly superior to the mentioned operators. This superiority in the proposed method can be attributed to the utilization of machine learning techniques, as machine learning models can perform edge detection with higher accuracy by learning edge patterns. On the other hand, comparing the two cases of R = 1 and R = 2 shows that increasing the neighborhood radius leads to improved edge detection results. In the R = 1 case, only pixels within a radius of one are considered for generating the feature vector of each pixel. In contrast, R = 2 increases the radius to 2 and employs a wider range of image features to describe the characteristics of each pixel. This property indicates that increasing the neighborhood radius can improve the accuracy of edge detection.

**Figure 6:**
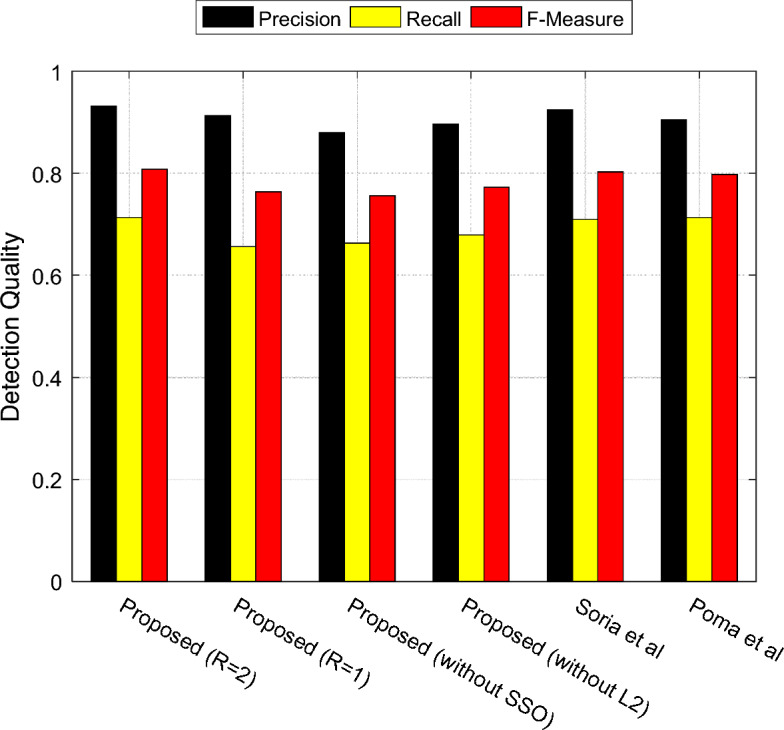
Performance of different methods in edge detection of color images from BSDS500 dataset based on precision, recall, and F-measure.

To provide a more accurate evaluation of the performance of the proposed method, precision, recall, and F-measure metrics can be utilized. In Fig. 6, the performance of various methods in detecting edges in color images from the BSDS500 dataset is shown based on these metrics.

According to the results presented in Fig. 6, the highest edge detection quality is achieved when using the proposed method (R = 2) for edge detection of images. The results show that in case of R = 1, the performance of proposed method is competitive with models presented by Soria et al.^13^ and Poma et al.^15^. On the other hand, if each one of SVM optimization or edge enhancement steps using SSO is ignored, the performance of the model considerably decreases. This shows that classical machine learning models such as SVM, if they take advantage of a precise hyperparameter configuration and output refinement, can still compete with deep learning methods in applications such as edge detection. The results indicate that the proposed method outperforms other compared methods in terms of precision, recall, and F-measure. The higher precision of the proposed method in edge detection implies that the identified edge pixels by this approach have a higher probability of being correct. Moreover, the higher recall in the proposed method indicates that our solution has been able to correctly extract a higher proportion of ground truth edge pixels. Finally, the higher value of F-measure confirms the higher quality of the edge-detected images obtained by the proposed method compared to other methods. This means that the outputs of the proposed method have higher TP values and, at the same time, lower FP and FN values. The F-Measure of the proposed method for (R = 2) was found to be 80.76%, which shows a minimum improvement of 0.53% compared to previous methods.

**Figure 7:**
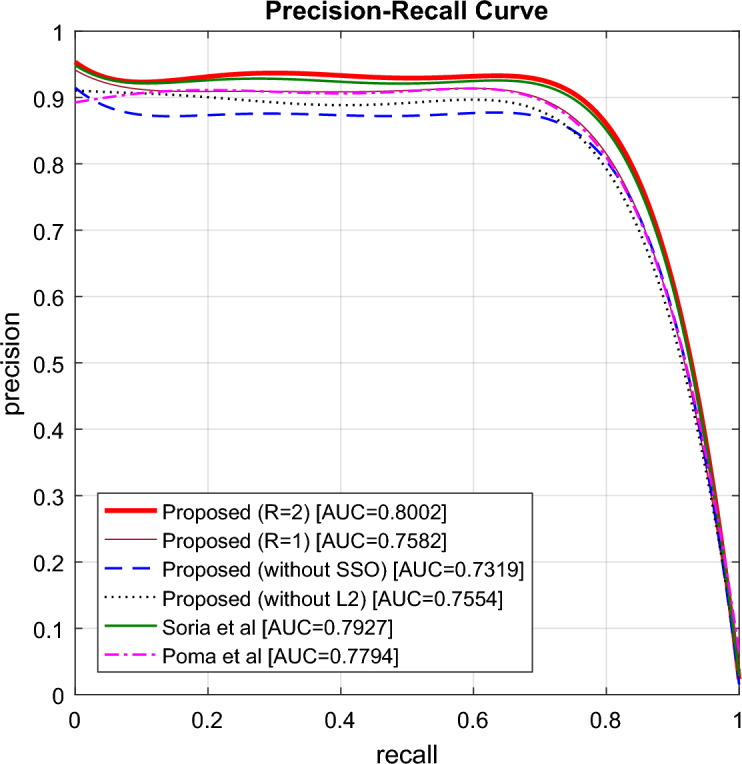
Precision–recall curve for different methods.

In Fig. 7, the precision-recall curves for different methods are plotted. In this figure, the horizontal axis represents different recall values, and the vertical axis represents precision values for different thresholds. This curve can provide a detailed insight into the performance of different methods in terms of accuracy in identifying edge pixels. Comparing the performance curves of different methods in Fig. 7 shows that the proposed method can achieve higher precision and recall values. According to this figure, the area under the precision-recall curve for the proposed method is 0.8002, and the closest approach to the proposed method is the solution proposed by Soria et al.^13^ with an AUC of 0.7927, which utilizes convolutional neural networks for edge detection. These results report an improvement of about 1% for AUC criteria and confirm the higher effectiveness of the proposed method in edge detection of color images.

**Figure 8:**
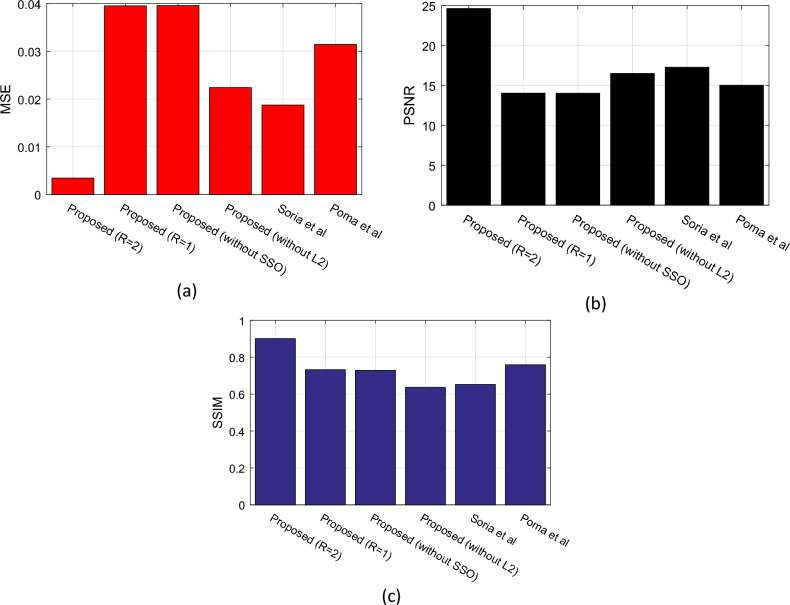
Performance comparison of different methods based on comparing edge detection results with ground truth edge image using (**a**) MSE, (**b**) PSNR, and (**c**) SSIM metrics.

Continuing with the evaluation of the proposed method, its performance based on the SSIM, PSNR, and MSE metrics is discussed. The results related to these metrics are plotted in Fig. 8. In Fig. 8a, the performance of different methods is compared in terms of MSE. This metric describes the squared difference between the ground truth images and the edge detection results. It is obvious that the goal of an edge detection method is to identify edge pixels with the least deviation from the background. According to Fig. 8a, the proposed method with R = 2 has the lowest average MSE for the test samples of the BSDS500 database. It should be noted that the ground truth and edge detection result images are binary, and each pixel can only have a value of 0 or 1. Based on these results, the MSE value in the proposed method is about one-fourth of the Soria et al.^13^ method (considered as the closest-performing method) and the proposed method can reduce MSE in edge detection by at least 0.0154. Additionally, Fig. 8b shows that the images resulting from edge detection by the proposed method have lower errors in terms of consistency with the ground truth images, leading to an increase in the PSNR metric. Finally, Fig. 8c confirms that the edge detection output of the proposed method has a higher structural similarity with the ground truth images, indicating the ability of our method to approximate the true edges of color images more accurately. This superiority in the proposed method can be attributed to the following two factors: firstly, the optimization of the SVM model by the SSO algorithm has allowed for a more accurate initial approximation of the input image edges based on this model. Secondly, the use of binary combinations of color channels to enhance edges by the SSO algorithm has facilitated the efficient utilization of color features in images for more accurate edge detection. The summary of the experimental results is presented in Table 2.

**Table 2:** Numerical values obtained from experiments.

Method	F-measure	Recall	Precision	SSIM	PSNR	MSE	Time(s)
Proposed (R = 2)	0.8076	0.7130	0.9311	0.9011	24.6274	0.0034	3.3155
Proposed (R = 1)	0.7633	0.6559	0.9128	0.7327	14.0296	0.0395	2.0568
Proposed (without SSO)	0.7559	0.6629	0.8791	0.7287	14.0225	0.0396	3.3056
Proposed (without L2)	0.7725	0.6790	0.8959	0.6366	16.4970	0.0224	0.0015
Soria et al.^13^	0.8023	0.7091	0.9237	0.6524	17.2685	0.0188	2.0235
Poma et al.^15^	0.7975	0.7130	0.9047	0.7588	15.0237	0.0315	1.5856

In Table 2, the performance of different methods is also compared in terms of processing time. It should be noted that the presented processing time values in this table are related to the testing phase using the trained model, and the training time (including model optimization for the proposed method) is not taken into account. Based on the results presented in Table 2, although the proposed method has better performance in identifying edges in color images, it requires a longer processing time, and this increase in processing time is due to the edge refinement step in the second step of the proposed method. Furthermore, these results are based on computations performed on graphics processors, and if the proposed method is executed on a CPU, the average edge detection time per image will increase to 335.595 s. Therefore, the increased computational burden resulting from the optimization step can be considered as one of the limitations of the proposed method, which should be addressed in future research.

**Figure 9:**
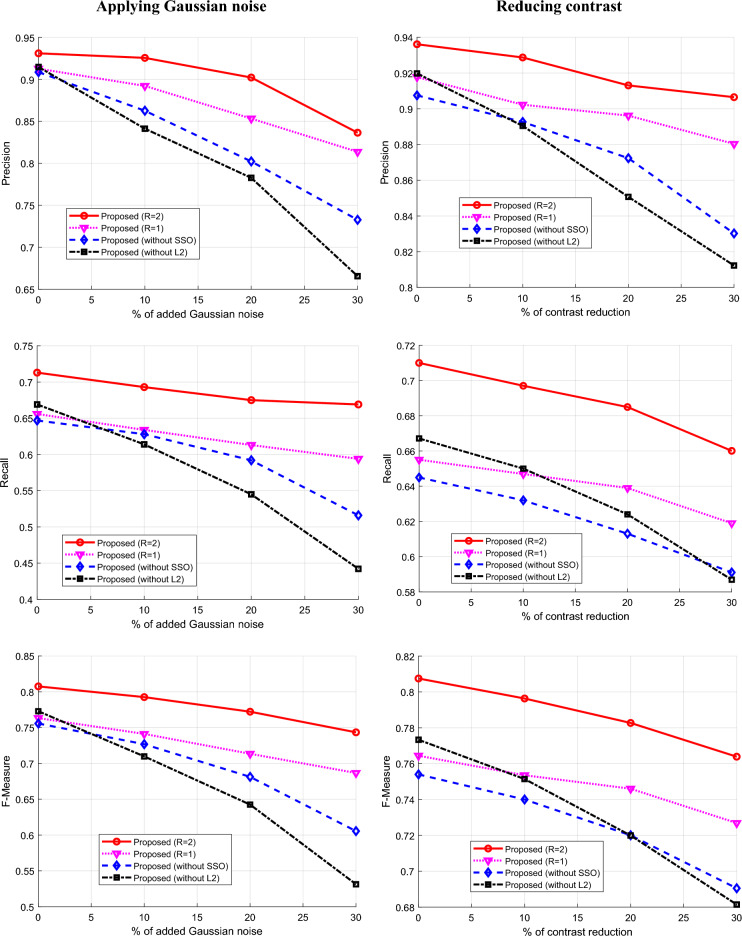
The performance of proposed method in case of (left) applying Gaussian noise and (right) reducing contrast in images in terms of (top) precision, (middle) recall and (bottom) F-measure.

In order to assess the performance of the proposed method more precisely, its flexibility in the condition of increasing noise and decreasing contrast in the images has been evaluated. Because the destructive effect of noise or low contrast are common in real-world images^30^. These results are presented in Fig. 9. In the left column of this figure, the results related to the effect of increasing noise on the performance of the proposed method in different conditions are given. Also, in the right column, the results related to assessing the effect of contrast changes are given. Each of these tests evaluated the performance of the proposed method in terms of precision, recall and F-Measure, the results of which are presented in the first to third rows of Fig. 9, respectively. Examining the performance of the proposed method in different situations shows its efficiency and flexibility in conditions of increased noise.

The results presented in Fig. 9 show that with a 30% increase in Gaussian noise in the input images, the edge detection quality of the proposed method (R = 2) decreases by only 6.4% in terms of F-Measure. On the other hand, if the edge enhancement process by SSO is ignored (Without L2 mode), this reduction will be 24.16%, which reports a more reduction compared to other operational scenarios of the proposed method. These results clearly show that the process of edge improvement by SSO in the proposed method has been able to significantly increase the resistance of this model against destructive effect of noise. On the other hand, examining the effect of contrast changes in Fig. 9 leads to similar results. These results show that 30% reduction of contrast, will result in only 4.36% reduction of F-Measure in the proposed method (R = 2). Meanwhile, if the SSO algorithm is removed in any step of SVM optimization or edge improvement, the F-Measure will drop by at least 11.7%. In this way, it can be concluded that the optimal performance of the proposed method and its resistance to the harmful effects of exposure depend on each step of SVM optimization and edge enhancement by SSO.

### Time complexity analysis and optimization considerations

This section analyzes the time complexity of the proposed method for edge detection in color images. The overall time complexity of the proposed method can be expressed as:

16 $${T}_{total}= {T}_{SVM}+ {T}_{SSO}+ {T}_{colo{r}_{processing}}$$

where, ${T}_{SVM}$ is the time complexity of training and classifying using the SVM model. In this case, utilizing an RBF kernel leads to a time complexity of $O\left(N * {S}^{2}\right)$ in the worst case, where N is the number of training samples and S is the number of support vectors. Also, ${T}_{SSO}$ is the time complexity of the SSO optimization algorithm. This depends on the chosen implementation but is generally considered to be $O(M * I * D)$, where M = 150 is the population size, I = 300 is the number of iterations, and D = 3 signifies the number of hyperparameters being tuned for the SVM model (typically C + , C-, and γ for the RBF kernel). Finally, ${T}_{colo{r}_{processing}}$ is the complexity of processing pairwise color layer combinations. Since the color system is RGB (L = 3), this complexity can be estimated as $O(W * H * L) = O(W * H * 3)$, where W and H are the image width and height.

Dominant factors and potential optimizations in terms of complexity of the proposed method, includes the following:

- Investigating the alternative optimization algorithms that exhibit faster convergence for tuning SVM hyperparameters.
- Exploring the potential of utilizing approximate optimization methods that can provide good results with lower computational demands.
- Designing the algorithm to leverage hardware acceleration capabilities (e.g., GPUs) for all processing steps of the proposed model (including training SVM model).

By analyzing the time complexity and exploring potential optimizations, the proposed method can be further improved for practical applications where computational efficiency is a concern.

## Conclusion

Edge detection in color images is one of the most commonly used image processing techniques, which, if an efficient method is provided, can enhance a wide range of processing tasks. However, edge detection in color images has received less attention compared to other applications. Therefore, this research focused on proposing an efficient method for edge detection in color images. The proposed method utilized the combination of SVM and the SSO algorithm to achieve this goal. The proposed approach performs edge detection in two levels. In the first level, an initial approximation of the edges in the grayscale image is created using an SVM model optimized by the SSO algorithm. In the second level, the SSO algorithm is employed to improve the detected edges. For this purpose, the edge pixels are displaced based on their compatibility with the pair-wise combinations of color layers in the image, using the standard deviation measure, in order to obtain an optimal displacement pattern for accurate edge detection of image regions based on their color features. The findings of the research demonstrated that the utilization of the SSO algorithm for optimizing the SVM model can be effective in achieving more accurate edge detection, leading to a reduction of edge detection error by approximately 5.17%. Furthermore, the adoption of the edge improvement strategy based on the SSO algorithm can decrease the edge detection error by approximately 3.51%. These results confirm that each of the techniques employed in the proposed method can have a positive impact on enhancing the accuracy of edge detection in color images. Additionally, the proposed model in this study was implemented using GPU-based parallel processing technology, and its effectiveness in accelerating the edge detection process was examined. The results of this analysis indicated that the utilization of this processing technology can increase the image processing speed by more than 100 times. Based on the results of the experiments, the F-Measure criterion of the proposed method for edge pixel detection in the BSDS500 image database was found to be 80.76%, which shows a minimum improvement of 0.53% compared to previous methods.

One of the limitations of the proposed method is the relatively high processing time in the second step (edge improvement). This increase in processing load is due to the extensive search space of the optimization problem for pixel edge editing. Although efforts were made in the proposed method to minimize this processing time by utilizing parallel processing techniques in GPU processors, real-time applications of color image edge detection still require faster solutions. Therefore, improving the processing speed in the second step of the proposed method can be a topic for future research. Additionally, in future studies, improving the performance of the proposed method can be attempted by replacing the SVM model with other learning models, such as CNNs.

## Data Availability

All data generated or analysed during this study are included in this published article.
